# Do Personality Traits Matter? Exploring Anti-drug Behavioral Patterns in a Computer-Assisted Situated Learning Environment

**DOI:** 10.3389/fpsyg.2022.812793

**Published:** 2022-05-20

**Authors:** Tien-Chi Huang, Yu-Jie Chen

**Affiliations:** Department of Information Management, National Taichung University of Science and Technology, Taichung, Taiwan

**Keywords:** anti-drug education, serious game, lag sequential analysis, K-12, Big Five personality traits

## Abstract

Drug abuse has been and continues to be, a common social issue worldwide, yet the efficiency of widely adopted sweeping speech for anti-drug campaigns has proven inefficient. To provide students with a safe and efficient learning situation related to drug refusal skills, we used a novel approach rooted in a serious learning game and concept map during a brief extracurricular period to help students understand drugs and their negative effects. The proposed game-based situational learning system allowed all students to participate simultaneously and individually in multiple scenarios of drug temptation posed by peers and classmates to practice responding and refusing drugs in school and community settings. Moreover, to explore whether different personality traits (such as the Big Five personality traits) result in different anti-drug responses, we used a serious game to conduct an anti-drug experiment on 53 junior middle school students aged 13–15. Each participant’s decision-making process was recorded in the serious game as behavioral patterns for lag sequential analysis (LSA). The outcomes revealed seven behavioral patterns including differentiation (D), acceptance (A), effective (ER) and ineffective responses (IR), effective (ES) and ineffective solution-seeking (IS), and failure to refuse (F). The GSEQ (Generalized Sequential Querier) which is a computer program for analyzing sequential observational data was used. The results indicated the following: (1) Neuroticism was performed at a relatively low level under the guidance of a concept map. (2) “Neuroticism” was associated with the lowest risk of accepting drugs. (3) Students with “openness to experiences” were at high risk of accepting drugs. (4) Almost all personality behavioral transition diagrams showed that failure to refuse (F) drugs was followed by inefficient seeking of help (SI) and inefficient refusal (RI). These findings provide reference points for designing adaptive anti-drug education programs.

## Introduction

Illegal drug abuse remains one of the world’s most serious social problems. The negative effects of drug abuse are harmful not only to personal health but also social development. Statistics from Taiwan indicate that the number of students abusing drugs in 2020 increased by 11% compared to the same period in 2019 (Ministry of Health and Welfare, Food and Drug Administration, 2020), highlighting the urgency of anti-drug education. In terms of the form of current anti-drug education in Taiwan, lectures using PowerPoint presentations are the most widespread way of promoting anti-drug education (Lucieer et al., 2016). However, research in Taiwan suggests that with such a form and content, Taiwanese students still lack knowledge of drug prevention (Chiu, 2016).

This highlights four key issues in traditional anti-drug education. The first is that learners fail to fully grasp the content with the current unstructured teaching materials (Baylor, 1999). Second, peer offers to use illegal drugs are a major test of courage and the ability to refuse (Newcomb et al., 1986). However, the “context of refusal” is missing in the traditional approach, which might lead to failure to refuse drugs when they are offered by peers. Third, behavioral responses in facing drug temptation have rarely been explored in past studies, and they are impossible to grasp through traditional approaches. In other words, instructors have difficulty understanding students’ behavioral responses or their decision-making process in an anti-drug context. Fourth, different people react differently to various drugs, but all students are exposed to the exact same learning materials in traditional settings. The antecedents that influence students’ anti-drug reactions were explored. The literature has confirmed the association between personality traits and smoking, finding that most drug users have low responsibility, high impulsivity, and high emotional vulnerability (Terracciano et al., 2008; Komarraju et al., 2011; Bajwa et al., 2017; Kim et al., 2019). However, few studies have investigated the differences in the selection of anti-drug scenarios based on personality traits.

To scrutinize the above issues, first, we chose a concept map (Novak et al., 1984) to introduce the structure of anti-drug knowledge to demonstrate the connection between old and new knowledge and to enhance meaningful learning. Second, we aimed to help students transfer what they learned in the classroom into a real anti-drug context in which peer influence is the most difficult aspect to control among the factors that contribute to adolescent drug use (Newcomb et al., 1986; Savin-Williams and Berndt, 1990; Cox et al., 2017). We adopted the theory of contextual learning (Brown et al., 1989) and developed a “serious game-based anti-drug learning system” to provide various kinds of scenario experiences, like peers encouraging or forcing the use of drugs and the choices they might face for guiding learners to “respond effectively by refusing drugs when offered [drugs] by their peers.” Third, we employed lag sequential analysis (LSA) based on users’ choices while utilizing the proposed serious game-based anti-drug learning system. By recording and analyzing students’ serial responses to the simulation context provided by the system, we investigated the decision-making process of successful and failure drug refusal. Fourth, we utilized the Big Five personality traits to examine students’ learning efficiency and behavioral patterns to identify the role of personality traits in anti-drug behavior.

According to the above research motivation, we propose five research questions:

1. Does anti-drug cognitive learning combined with a concept map helps improve the learning effect of anti-drug education?
2. What do participants think about the proposed anti-drug approach (the concept map and the serious game-based learning system)?
3. What is the sequence pattern of students’ behavior in the context-oriented anti-drug learning experience?
4. Are there any differences in the effectiveness of anti-drug learning among students with different personality traits?
5. Do learners with different personality traits have different sequential patterns of anti-drug behavior?

## Literature Review

### The Personality and Anti-drug Pattern

Integrating the health belief model (Rosenstock et al., 1988), the theory of reasoned action (Fishbein et al., 2002), and social cognitive theory (Bandura, 1977), Fishbein and colleagues proposed an integrated theoretical model to be applied to health-related behavioral prevention. Among all the latent external variables, the personality trait is a fairly stable variable (Roberts et al., 2006) that requires the cooperation of educators to teach students as per their aptitude.

The development of personality trait theory started from the trait school in which Allport and Odbert (1936) proposed more than 4,000 possible trait terms. Cattell (1949), based on the terms of traits and behaviors proposed by Allport and Odbert (1936), divided them into 16 personality factors through factor analysis, significantly reducing and categorizing the types of personality factors, which laid the foundation for the five-factor model of personality (FFM). Following Cattell’s (1949) study, Tupes and Christal (1992) identified five major factors of personality differences from the test results. Subsequently, scholars again validated the FFM of personality and confirmed that it agreed with the personality classification approach. The findings did not differ much from the development, structure, and reliability of Tupes and Christal’s tests. Therefore, this classification approach has been generally recognized and applied in practice. The final five personality factors that have been developed and compiled include agreeableness, conscientiousness, extroversion, neuroticism, and openness to experience, as shown in the following Table 1.

**Table 1:** Description of the five personality traits (John et al., 2008).

Dimension	Meaning	Features
Agreeableness (A)	The degree of interpersonal orientation from compassion to hostility in one’s thoughts, feelings, and behaviors	Kind-hearted, tolerant, and helpful
Conscientiousness (C)	The degree to which the individual is organized, persistent, and motivated in the pursuit of goals	Reliable, conscientious, responsible, and ambitious
Extroversion (E)	The intensity with which the individual enjoys contact with others and participates in social activities	Optimistic, active, enthusiastic, and talkative
Neuroticism (N)	The extent to which individuals feel…	Nervous, emotional, insecure, and depressed
Openness to experience (O)	The extent to which individuals pursue and explore new experiences and interests	Curious, creative, imaginative, and inventive

Past research has examined the relationship between many personality traits and learning variables: conscientiousness affects the stability of test scores (Chamorro-Premuzic and Furnham, 2003); neuroticism can easily cause test or study anxiety due to emotional instability, which impairs learning performance (Poropat, 2009); and extroversion is more focused on interpersonal interactions and is prone to attention deficits (Rolfhus and Ackerman, 1999; Bidjerano and Dai, 2007). Self-discipline, strong will, ambition, and high frustration resistance positively affect learning outcomes (Tziner et al., 1991). Some studies have also explored the personality traits of drug users. Empirical studies suggest that those with drug use disorders have similar personalities (Zilberman et al., 2018): Most drug users have low conscientiousness, high impulsivity, and high emotional vulnerability (Terracciano et al., 2008). Furthermore, sensation-seeking, as one of the predictors of drug abuse, is a personality trait (Donohew et al., 1994; Martin et al., 2002). In a study on anti-drug education, D’Silva and Palmgreen (2007) proposed that sensation-seeking personality traits are predictive of anti-drug public service announcements (PSAs). In sum, there is a correlation between drug use behaviors, responses to anti-drug messages, and personality traits. The occurrence of drug use behavior implies that the initial exposure to drugs did not result in successful refusal—and instead the acceptance of drugs—and therefore, refusal behavior may also be related to personality traits. However, little research has been conducted to examine the context of students’ drug refusal and their personality traits in drug refusal behavior. Thus, we designed this study to identify the anti-drug selection process of different personality traits through behavioral sequence analysis.

### New Approach of Anti-drug Education: A Concept Map and Situated Interaction System

The United Nations Office on Drugs and Crime (UNODC, 2004), in its report on education about drug abuse prevention in schools, states that anti-drug education aims to achieve the health goals of preventing drug use and abuse through formal and informal health education programs, which provide youth with knowledge about drugs and life skills, and enable youth to face and deal with current situations without falling into the arms of drugs when necessary.

In 2000, the Ministry of Education incorporated anti-drug education into the 9-year curriculum for elementary and middle school students. Students are taught health development, life skills, and interpersonal relationships in elementary school, and are subsequently introduced to the basics of tobacco, alcohol, betel nuts, and addictive drugs. From middle school through high school, students are taught information and regulations on addictive drugs through health, physical education, and military training programs to acquire the concept and knowledge of drug prevention. Moreover, as early as 1990, the Ministry of Education (2022) implemented the “Chun Fai Program” in schools at all levels; it had five major areas of prevention-related content: drug abuse, smoking, alcohol abuse, betel nut chewing, and AIDS. In recent years, the Echinacea Campaign has been used to continue the anti-drug goals of the Chun Fai Program in the hope of promoting an anti-drug culture among students and spreading correct anti-drug concepts.

Anti-drug education can be divided into different dimensions, such as memory, understanding, learning transfer, and application. In the past, anti-drug education has mostly been conducted through thematic lectures, law education campaigns, anti-drug knowledge learning, and anti-drug propaganda on campus (Lucieer et al., 2016), which may help understand drug knowledge. In anti-drug education, students are not only the receivers of information but also the processors. When information is delivered, teaching materials that provide a structure of knowledge allow students to think deeply or connect to past experiences (Baylor, 1999). The concept map—an illustration that presents conceptual relationships—has been suggested as a teaching and learning strategy that can help students to integrate new and old knowledge. Lectures with concept map teaching are not beneficial for knowledge acquisition and lowering cognition load (Chen et al., 2011; Abediny and Tabibi, 2012; Aliyari et al., 2019). However, in practical and meaningful learning, the concept map group performed significantly better (Haugwitz et al., 2010; Chen and Wu, 2015; Schroeder et al., 2018). In the present study, we intended to explore the pattern of students’ high-level judgments about anti-drug behavioral choices; we thus incorporated concept maps into anti-drug instruction.

According to a study by the Foundation for a Drug-Free World, adolescents’ motivations for first-time drug use are mainly to fit in with their peers, curiosity, escape, and stress relief (Huang et al., 2013); up to 36.1% of illicit drugs came from friends. Moreover, the psychological state of desire for peer recognition easily drives adolescents to use illicit drugs (Huang et al., 2013). The above study highlights that the most important factors influencing adolescents’ illicit drug use are peers and personal psychological resistance to stress and that students’ drug knowledge alone may not be sufficient to effectively generate appropriate anti-drug behaviors in peer pressure situations. More specifically, cognitive learning activities alone in anti-drug education might not be able to represent the peer pressure encountered in real anti-drug situations or to improve an individual’s ability to successfully refuse drugs.

Situated learning, derived from cognitive-contextual theories (Brown et al., 1989), is a strategy that advocates for meaningful learning through changes in the teaching environment; the instructor constructs real or simulated situations in which learners interact with the environment to develop the pattern and meaningful learning and to understand how to apply what has been learned (Errington, 2003; Tanggaard, 2007; Yetik et al., 2012; Huffman et al., 2016). Thus, situated learning constructs knowledge and skills for learners to interact with and explore a situation, thereby bridging the gap between theory and practice (Meldrum, 2011). Further, the more similar the situational design is to real-life cases, and the higher the degree of simulation of life situations, the more that learners can adapt their acquired knowledge and skills to the real world. In other words, the closer a situation is to reality, the better the learning transfer effect.

In conclusion, to help learners enhance anti-drug learning outcomes, we first constructed concept map-based, anti-drug learning materials in cognitive terms; in contextual terms, we used a Web-based system to create a dynamic drug exposure situation in which students experienced an “animated dialogue-based interactive situation” and chose their own responses to advance the plot and explore the interactive anti-drug story.

## Research Methods

### Participants

To answer these four questions, we conducted an anti-drug experiment on 53 junior middle school students aged 13–15. The school is in East District, Taichung, in middle Taiwan. In 2016, the Eastern District ranked 9th among the 30 districts in Taichung City, with an average annual household income of US $30,518 and a median of US $20,888. The student population is composed of 99% Han Chinese and less than 1% of Aborigines. The proportion of male and female subjects is similar (male = 48.5%, female = 51.5%), of which 87.1% participated in anti-drug activities. Public lectures (47.2%) and educational films (35.0%) were the most common types of anti-drug activities.

### Instruments

Our research methods included the scale of the Big Five personality traits, a cognitive test (I), a basic test of drug abuse, a. cognitive test (II) of drug abuse prevention and control, and a cognitive test (III). The tests and scales are described in detail below. Further, to demonstrate the difficulty of the test, we used the following formula: P = R/N, in which P represents the difficulty index of the question. R is the number of people who answered the question, and N is the number of all subjects (Brown et al., 2013).

#### Scale of the Big Five Personality Traits Data

We referred to the simplified version of the NEO Five-Factor Inventory (NEO-FFI) proposed by Körner et al. (2015). The scale provides a concise measure of the five basic personality factors and includes five dimensions—agreeableness, conscientiousness, extroversion, neuroticism, and openness—with a total of 30 items. Each question is scored on a five-point Likert scale, ranging from (1) *strongly disagree* to (5) *strongly agree*.

We utilized the Big Five scale of the personality test (agreeableness, conscientiousness, extroversion, neuroticism, and openness to experiences) before the experiment.

#### Cognitive Test I: Basic Test of Drug Abuse (Pre-test)

The purpose of this test was to measure prior knowledge of drug abuse. The content of the test was based on the question bank of the Ministry of Education’s website on drug abuse prevention and the Ministry of Justice’s anti-drug website. The content category of the test is the foundational knowledge of drugs (traditional and emerging drugs), methods of judging and rejecting drugs, and legal norms. We adopted a pen-and-paper test with a total of 15 questions (including yes/no questions), fill-in-the-blank questions, and multiple-choice questions; the participants were given 15 min to take it. Five points were given for each correct answer to fill-in-the-blank questions and Q&A. Finally, the total score (out of 100 points) was calculated. The difficulty (P) of the full test questions ranged from 0.29 to 0.92, with an average value of *p* 0.65.

#### Cognitive Test II (Post-test 1)

The purpose of the test was to evaluate students’ scores after 2 weeks of learning (teaching unit: what are drugs, emerging drugs, traditional drugs, and ways of judging and rejecting drugs). The types of questions were the same as those on the Basic Test of Drug Abuse. There were 17 questions in total. The difficulty (P) of the entire test ranged from 0.37 to 0.98, with an average value of *p* 0.75.

#### Cognitive Test III (Post-test 2)

The purpose of this test was to assess students’ scores after learning was complete (what are drugs, emerging drugs, traditional drugs, ways of judging and rejecting drugs, and laws and regulations related to drug hazard prevention and control). The types of questions were the same as those on the Basic Test of Drug Abuse. The difficulty (P) of the entire test was between 0.26 and 0.96, with an average value of *p* 0.64.

#### Interview

To understand the students’ thoughts on the anti-drug education course in this study, we held a 5-min, semi-open-ended interview with each student after the experiment. The interviews included three dimensions: learnability, interestingness, and future behaviors.

### Experimental Process

**Figure 1:**
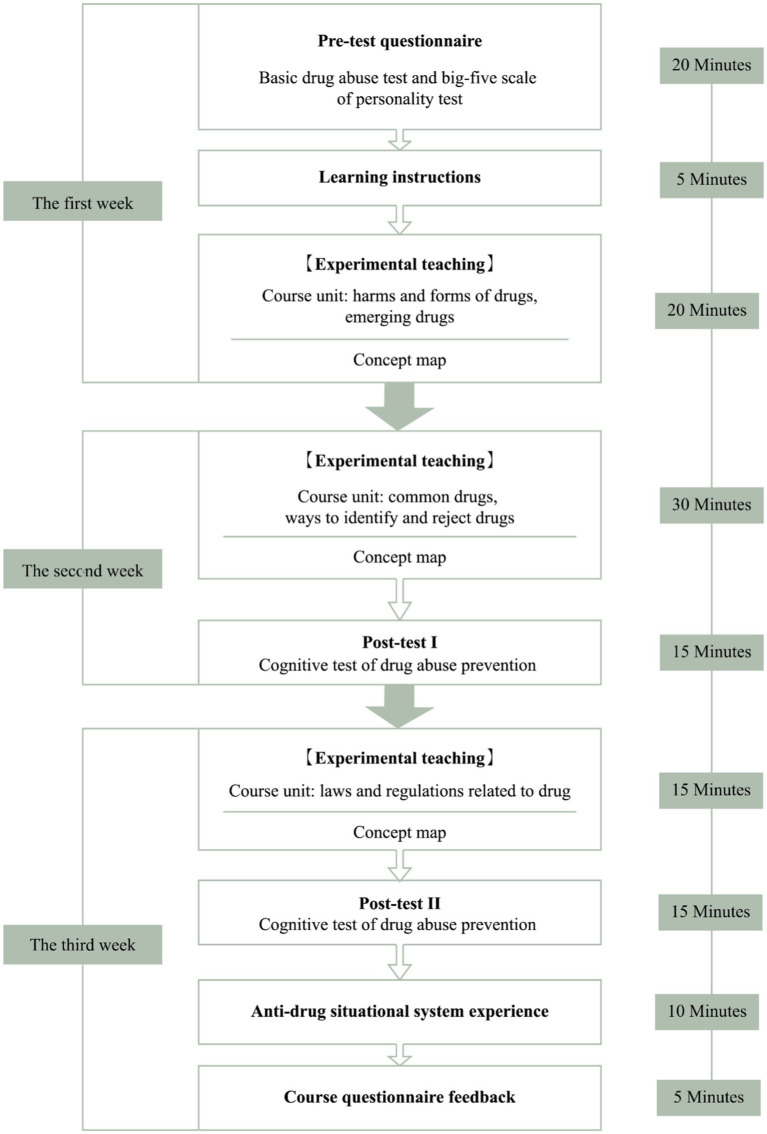
Experimental flow chart.

The experimental process was divided into two stages. The first stage included asking students to take the Basic Test on Drug Abuse with the scale of the five personality factors, and then complete the teaching experiment. The students took part in a 3-week teaching experiment and assisted learning with concept map teaching materials. After the second week of class, the students were given Cognitive Test I on drug abuse prevention, and after the third week, the students were given Cognitive Test II on drug abuse prevention. During the second stage, after the students completed the anti-drug teaching experiment, they continued to engage in the serious game-based situational learning system. As mentioned above, the purpose of the system is for students to experience the process of refusing drugs in an authentic interaction. The experimental process is shown in Figure 1 below:

#### Experimental Teaching Materials

The content of the textbook (compiled by us) on drug prevention and the control “concept map” used in this study were based on the health education content from the resource website of the Ministry of Education on drug abuse prevention and the anti-drug website of the Ministry of Justice.

**Figure 2:**
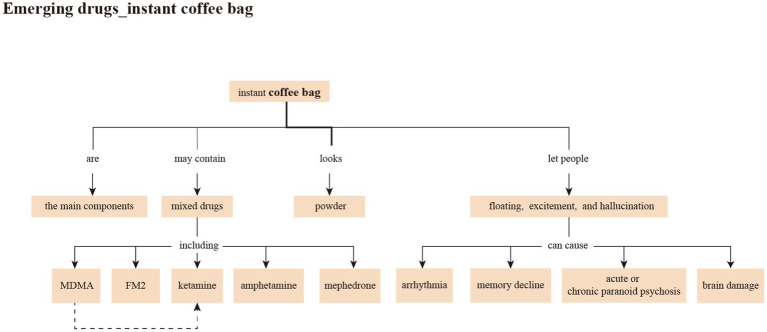
Schematic diagram of conceptual map presentation.

- Learning objectives: To guide students to fully understand the relationship between the key concepts of anti-drug education through the concept map and to steadily internalize the teaching materials.
- Content of teaching materials: The content of teaching materials includes basic drug knowledge and laws. After planning all the content, we invited several students to discuss the content with two anti-drug education experts and then revised the content according to their suggestions.
- Presentation of teaching materials: We designed the concept map teaching materials according to the Novak concept map structure. By learning top-down (abstract to concrete) hierarchical concepts, we helped the students to connect new knowledge with their previous experiences. The attached pictures provide an explanation and a detailed introduction, as displayed in Figure 2 below.

#### The Proposed Serious Game-Based Situational Learning System

**Figure 3:**
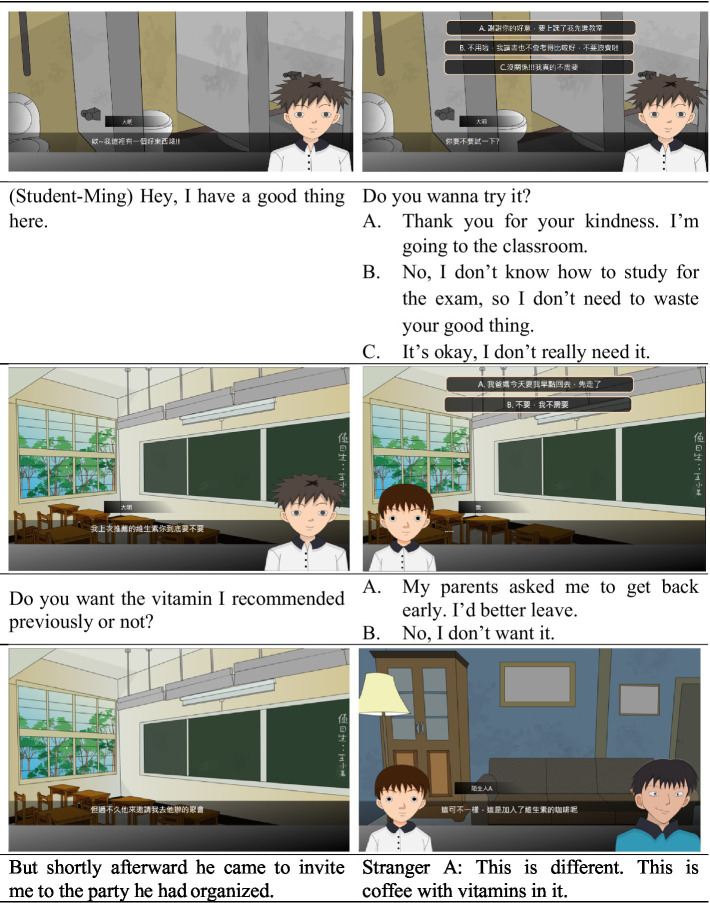
An example of a dialog and choice items in the system.

The serious game-based situational learning system for anti-drug education consists of multiple storylines of exposure to drugs. Visually, the comic-style conversation demonstrates the scenarios of drug temptations and coercion from peers and friends in the community and on campus. In these conversation scenarios, learners can choose to respond, either rejecting or accepting the drug upon being presented with an offer, which leads to different results. In other words, the system allows learners to experience various scenarios of exposure to drugs that are similar to real-life situations. An example of a dialog in the system is outlined below in the following Figure 3.

Through fictional experiences like those portrayed above, the students come to sense the peer pressure they might face when refusing drugs. They can choose how to respond to each situation (through multiple-choice questions). It takes around 5–10 min to complete the situational experience. Depending on their choices, students enter various branches of the plotline, eventually leading to success or failure to turn down drugs. To make the dynamic scenario as genuine as possible, the system exacerbates scenarios of peer pressure based on students’ choices; the more the students try to refuse drugs, the higher the peer pressure becomes. After a round of situational experience, the system will guide to refuse drugs according to the decisions made by the student in the situational story.

#### Lag Sequential Analysis

**Figure 4:**
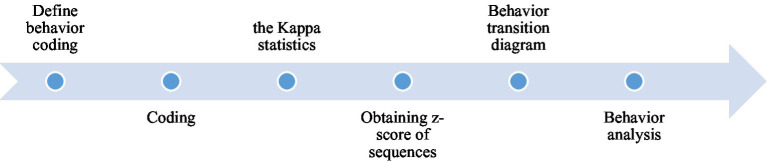
The procedure of lag sequential analysis (LSA).

In this serious game-based situational learning system, each participant’s decision-making process was recorded automatically by the system, and the log data were exported as personal behavioral patterns to code and analyze. The analytical flow chart is presented in Figure 4. First, we reviewed and identified all recorded behaviors and coded the results. Next, two experts discussed the appropriateness and saturation of behavioral codes and reached a consensus. The code outcomes revealed seven behavioral patterns, including differentiation (D), acceptance (A), effective (ER) and ineffective responses (IR), effective (ES) and ineffective solution-seeking (IS), and failure to refuse drugs (F; Table 2).

**Table 2:** Coding table of the scenarios.

Code	Stage	Definition	Example
D	Distinguishing	The student notices unusual situations.	This looks like the emerging drug introduced on TV! It would be better to leave while I can.
A	Accepting	The student accepts invitations and instigations from friends or compromises with a menacing situation.	Everyone is eating, and I will take a bite too.
RE	Responding effectively	The student can skillfully evade or successfully refuse friends’ invitations and instigations.	If I smoke only to prove that I can, then I am too indecisive.
RI	Responding ineffectively	The student fails to calm down and provokes conflict, which results in unsuccessful rejection.	I get angry at being called a coward. I need to make a good appearance.
SE	Seeking assistance effectively	The student seeks outside assistance (teachers and family members) for the events they encounter.	Seek help from teachers immediately.
SI	Seeking assistance ineffectively	The student seeks outside assistance (peers) for the events they encounter.	Call classmates for help.
F	Failing to refuse drugs	The student uses drugs either on his/her initiative or is forced to do so.	I do not want to ruin this relationship. Just drink it.

Three experts coded the recorded behavior and confirmed the coder’s reliability. Next, sequence analysis was used to construct the transformation of each group of behavior sequences. In addition, to understand the differences in the selection context of students with different personality traits, we performed a sequence analysis of students with different personality traits to determine the transformation of their behavioral sequence.

## Results and Discussion

### Structured Teaching Materials Can Help Improve Students’ Learning Effectiveness Regarding Anti-drug Knowledge

To answer question 1, “Does anti-drug cognitive learning combined with a concept map help improve the learning effect of anti-drug education?” we administered three tests—including an anti-drug cognitive pre-test and post-tests I and II—in succession to the participants. A dependent sample *t*-test indicated a significant difference (*N* = 53, *t* = 2.968, *p* < 0.01) between the pre-test (*M* = 67.64, *SD* = 11.12) and post-test I (*M* = 75.13), meaning that presenting concept maps improved the students’ learning effectiveness regarding anti-drug knowledge. In other words, with the assistance of concept maps, the students understood concepts tackled in learning activities and improved their academic performance; this outcome is in line with previous concept map-related research (Wu et al., 2012; Hwang et al., 2019; Zubaidah et al., 2020; Chang et al., 2022).

However, after the third week of the anti-drug course, the average score of post-test II declined (*M*_post1_ = 75.13, *SD*_post1_ = 14.73; *M*_post2_ = 72.34, *SD*_post2_ = 14.11), but this represented no significant difference from that of post-test I. According to the interview results, repetitive learning of fairly simple content and too many tests may have reduced the students’ concentration and patience as the number of classes increased, resulting in low learning motivation and affecting retention. This may be the reason for the drop in scores.

### Students With Neurotic Traits Had the Lowest Performance in Anti-drug Learning Effectiveness

Descriptive statistics for anti-drug learning effectiveness (post-test II) of participants with different personality traits are described in the table below (Table 3). Students who received concept maps to aid learning performed differently according to their personality traits. The mean scores for participants who scored high in neuroticism were lower.

**Table 3:** Descriptive results of the Big Five scale.

Personality trait	Mean	SD	Number
Openness to experiences (O)	67.35	13.50	34
Agreeableness (A)	63.39	13.69	31
Neuroticism (N)	57.19	13.41	16
Conscientiousness (C)	66.25	16.37	20
Extroversion (E)	68.33	14.55	18

A *post-hoc* test of ANOVA further indicated that the learning effectiveness scores for participants with high levels of openness and agreeability were significantly higher than those with greater levels of neuroticism (Table 4). Neuroticism refers to a broad range of negative emotionality like worry and depression (Luciano et al., 2018). Previous studies suggest that neurotic students who experience anxiety, negative emotions, and self-denial tend to withdraw from learning (Allik and McCrae, 2002; Studer-Luethi et al., 2012). Compared to other traits, neuroticism includes a lower likelihood of persisting in learning when encountering difficulties. This trait is positively correlated with academic procrastination (Bäulke et al., 2021) and negative affective priming (Robinson et al., 2007) and is also associated with poorer mental health (Strickhouser et al., 2017). Therefore, similar to other studies on learning content, students with higher levels of neuroticism had poorer performance in anti-drug learning than those with higher levels of other traits when using the same structured learning materials.

**Table 4:** ANOVA of learning effectiveness among different learning strategies and personality traits.

Trait		MD (I–J)	SE	Significance	95% CI	
(I)	(J)				Lower bound	Upper bound
O	A	−0.1905	4.8371	0.969	−9.869	9.488
	N	14.9206^*^	5.7006	0.011^*^	3.514	26.327
	C	5.2679	5.9447	0.379	−6.628	17.163
	E	5.7792	5.3255	0.282	−4.877	16.435
A	N	15.1111^*^	6.0329	0.015^*^	3.039	27.183
	C	5.4583	6.2641	0.387	−7.076	17.993
	E	5.9697	5.6798	0.298	−5.396	17.335
N	C	−9.6528	6.9526	0.170	−23.565	4.259
	E	−9.1414	6.4311	0.160	−22.010	3.727
C	E	0.5114	6.6485	0.939	−12.792	13.815

*p < 0.05.

### Qualitative Feedback From the Participants

Regarding the feedback from junior high school students who took part in the anti-drug course (see the table below), the students believed that the learning process and teaching materials of the experiment were interesting and that they had learned more about drugs and methods of prevention. Further, some students mentioned that with the assistance of the structured teaching materials, they could more quickly memorize and understand knowledge and established alertness for offers of drinks from strangers.

Learnability-

*Learning in order and by category helps impart knowledge*.

*There are plenty of takeaways and these can be quickly memorized*.

*The presentation of text boxes and illustrations helps with memory retention*.

*I have a better understanding of the drug categories and their dangers*.

*Categorized teaching allowed us to more easily understand the drug categories*.

*I learned about the effects of drugs and prevention methods*.

*The course made it easy to understand how to identify different drugs*.

*The course helped me understand anti-drug knowledge quickly*.

*Using a layered approach (concept maps) can facilitate understanding*.

Interestingness-

*The course is far more interesting than the previous ones, and I have learned a lot*.

*The course was interesting, and I did not get bored*.

*The course was lively, which held our attention*.

*I think it is great and I expect similar courses next time*.

Future behaviors-

*Stay away from drugs*.

*Do not take drugs*.

*I will not be deceived*.

*Never try drugs*.

*I know what should not be consumed and how to prevent emerging drugs from entering my life*.

### Sequential Analysis of Anti-drug Situational Experiences

To analyze the students’ reactions in anti-drug scenarios, we employed LSA to examine their experiences with the anti-drug scenario system. All behavioral codes were determined based on the anti-drug scenario. We explored seven behaviors related to drugs, including distinguishing, accepting, responding effectively, responding ineffectively, seeking assistance effectively, seeking assistance ineffectively, and failing to refuse drugs. To understand students’ reactions to their peers, we further divided “responding” and “seeking assistance” into responding effectively and ineffectively and seeking assistance effectively and ineffectively, respectively. “Failing” means that a student failed to refuse; the student in the scenario chose to accept and use drugs, resulting in the end of the experience.

**Figure 5:**
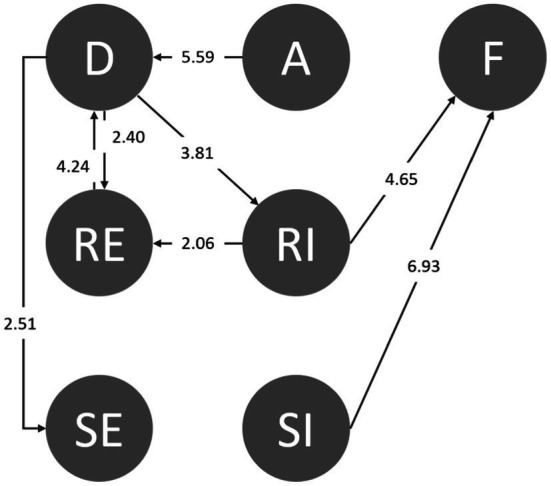
The behavioral transition diagram after using the serious game-based situational learning system.

We collected 95 sets of codes (a total of 20,550 behavioral codes) from the students during the period of using the serious game-based situational learning system. We then inferred the behavioral transition diagram *via* LSA using GSEQ5.1 software. The results of frequency transition and the adjusted residuals (z-score) of each set were produced. The sequential behaviors with the z-scores above the significance level of 1.96 were transformed into behavioral transition diagrams. Figure 5 shows the significance sequences. The arrow indicates the direction of transfer for each sequence.

The sequential analytical outcomes of six major behaviors are as follows: D➔RE (distinguishing➔responding effectively), D➔RI (distinguishing➔responding ineffectively), A➔D (accepting➔distinguishing), RE➔D (responding effectively➔distinguishing), RI➔F (responding ineffectively➔fail), and SI➔F (seeking assistance ineffectively➔failing).

First, the behavioral pathway of D➔RI suggests that students who noticed abnormalities in the environment as well as in their friends had taken certain actions but could not effectively reject their peers or friends. This means that when the students’ friends instigated the use of drugs or demanded that they use drugs, if they did not respond appropriately and effectively, they would still have to use the drugs despite understanding the abnormalities of the request. Thus, the strategies and techniques for refusing drugs require training and exercise. In addition, when students are aware of unusual situations and dangers, seeking help directly and effectively (D➔SE) can avoid the risk of being exposed to danger.

Such associations can also be found in D➔RE and RE➔D. Both pathways suggest a bidirectional association between students’ “effective rejection after distinguishing” and “re-distinguishing after effective rejection.” This means that the students tended to remain alert against danger. Although they effectively rejected an offer, the students were still alert. Distinguishing and effective rejection could produce a reinforcing effect. After accepting a friend’s invitation, the students could still recognize the drugs. Although they could not respond effectively at the beginning, they finally and effectively rejected the drugs after adjusting (A➔D➔RI➔RE). However, pathways RI➔F and SI➔F indicate that once the distinguishing was complete if the students moved toward “responding ineffectively” (RI), they would be forced to accept drugs in the same way as after “seeking assistance ineffectively” (SI).

### Behavioral Sequences Among Students With Different Traits

To determine the behavioral differences among students with different traits, we categorized the students into the following five groups based on their scores on the Big Five inventory: openness to experiences, agreeableness, neuroticism, conscientiousness, and extroversion. Openness to experiences includes the tendency to be attracted to external novelties and a willingness to explore. Agreeableness is characterized by empathy and altruism and the ability to convey warm, emotional interactions. People who exhibit a high degree of neuroticism are prone to stress or cannot easily keep calm when encountering stress. Conscientiousness is characterized by concentration on activities but being too cautious and fearful, thus limiting the possibility of activities. People high in extroversion are good at participating in social activities and are sensitive to potentially nearby rewards. We examined the behavioral patterns of these five groups of students one by one to explore their characteristics.

#### Agreeableness

**Figure 6:**
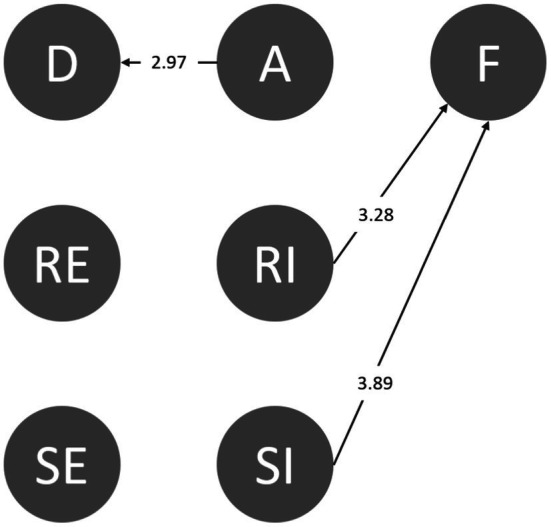
Behavioral transition diagram of agreeableness.

Agreeable people get along with others, do not like to argue, and are willing to compromise, abandoning their stances and needs to get along smoothly with friends. According to Figure 6, all the students in this category failed after rejecting ineffectively (RI➔F). In addition, people with high agreeableness more easily trust others and are less defensive. Based on the above, agreeable students are not good at refusing or suspecting others. Further, after learning, students with high agreeableness could still detect danger in the environment (A➔D) and sought assistance, even after having accepted an invitation from friends. However, their attempts were ineffective (SI➔F; Figure 6).

#### Conscientiousness

**Figure 7:**
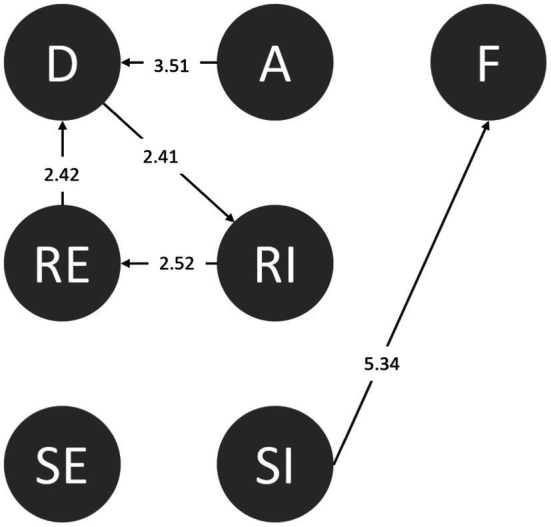
Behavioral transition diagram of conscientiousness.

From some perspectives, conscientious people are more organized and tend to consider the consequences of an action before performing it. After students accepted invitations from their friends, they could consciously identify danger (A➔D). Alternately, when they considered the negative consequences of accepting friends’ instigations, they attempted to reject this, even though it was ineffective (SI➔F; Figure 7).

#### Extraversion

**Figure 8:**
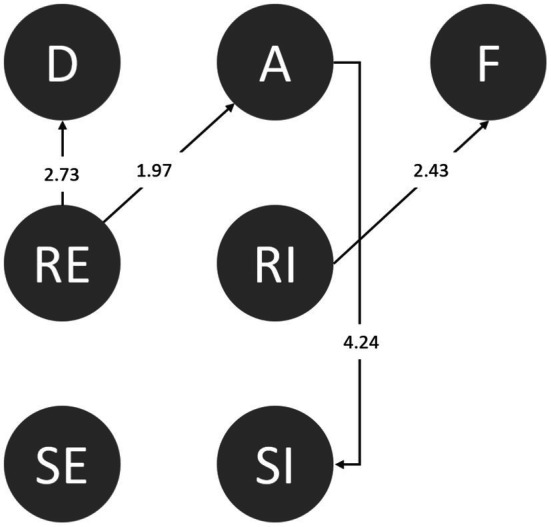
Behavioral transition diagram of extraversion.

Extraverts are good at making friends and participating in activities and enjoy the company of others. Extraverted students directly went from responding ineffectively to failure (RI➔F). Even though students declined their friends effectively, they were still likely to accept invitations (RE➔A). The inconsistency in their behavior suggests that the behaviors of extraverted students are hard to predict or that they were confused and could not make accurate judgments. Even so, after effective rejection, the students were still alert to their surroundings (RE➔D; Figure 8).

#### Openness to Experiences

**Figure 9:**
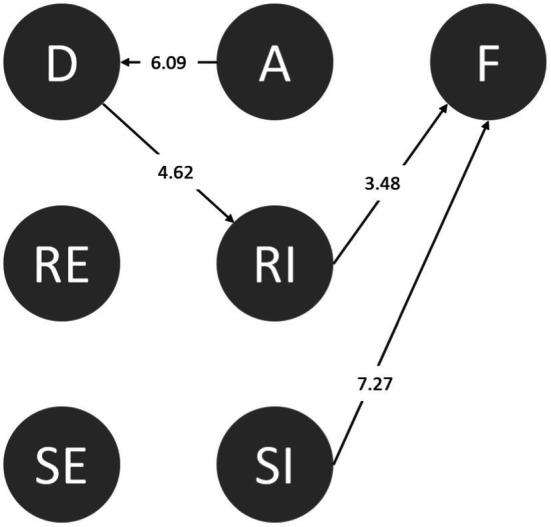
Behavioral transition diagram of openness to experiences.

People high in openness to experiences like to be exposed to new things and try different activities. All students in this category sought assistance ineffectively and were forced to use drugs (SI➔F). However, students who accepted their friends’ invitations noticed the abnormalities in their surroundings (A➔D). Despite this, they could not effectively reject their friends and were forced to use drugs (D➔RI➔F; Figure 9).

#### Neuroticism

**Figure 10:**
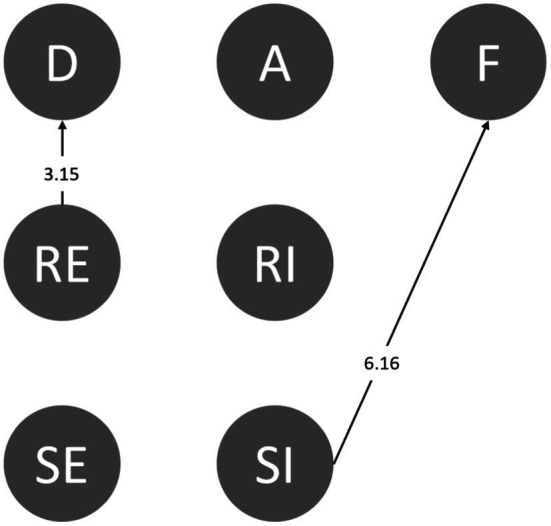
Behavioral transition diagram of the two sets of neuroticism.

Neurotic people are less likely to be calm and composed in the face of pressure and cannot regulate and respond to emotions as well as others. After effective rejection, students with high levels of neuroticism were still alert to the abnormalities in their surroundings (RE➔D), but similar to others, they failed to refuse drugs after seeking assistance ineffectively (SI➔F; Figure 10).

## Conclusion

Drugs destroy people’s health and wealth, harm families and society, and damage a country’s competitiveness. To prevent the scourge of drugs, efficient anti-drug education must be implemented immediately. There have been many anti-drug activities at the current stage, but most are sweeping speeches that do not foster learning efficiency, especially in terms of cognitive improvement and refusal skills.

Hence, we began with the design of a concept map to be used in a brief extracurricular period to help students understand drugs and their negative effects. Additionally, to provide students with a safe, efficient learning situation related to drug refusal skills, we developed a serious game-based situational learning system that allowed all students to participate simultaneously and individually. Multiple scenarios of drug temptations posed by peers and classmates were demonstrated, and the students could practice responding and refusing in school and community settings. From this experience, the students understood the need to resist peer pressure to successfully reject drugs.

To provide practical suggestions for educators and to identify potentially high-risk students, we adopted the Big Five personality traits, which are relatively stable predictors. According to the results of the teaching experiment, cognitive performance for students who were identified as having neuroticism remained relatively low under the guidance of the concept map, which is consistent with the findings of some previous studies (Bäulke et al., 2021). Based on the results of LSA statistics, almost all the personality behavioral transition diagrams—including seek help inefficiently ➔ failure (SI➔F) and refuse inefficiently ➔ failure (RI➔F)—achieved a significant level. This indicates that seeking assistance efficiently and refusal skills are crucial for the successful refusal of drugs. Moreover, students with high neuroticism were not at high risk as we had thought according to a significant pattern, signaling that they can respond effectively and differentiate (RE➔D). Actually, among the five personality traits, “neuroticism” was associated with the lowest risk of accepting drugs.

In contrast, the significant behavioral pattern (A➔D➔RI➔F) associated with “openness to experiences” was the most concerning, further implying that an “open” personality might also be open to drugs. People with open personality traits tend to actively participate in novel social and physical situations of social exploration, including making new friends, participating in discussions on unfamiliar topics, and voluntarily joining new organizations (Hirsh et al., 2009). In addition, “openness,” “agreeableness,” and “conscientiousness” all have significant patterns beginning with “accept drugs” (A). Contrarywise this, the “extroversion” presented a chaotic behavioral sequence with significant patterns, both for RE➔D and RE➔A➔SI.

**Figure 11:**
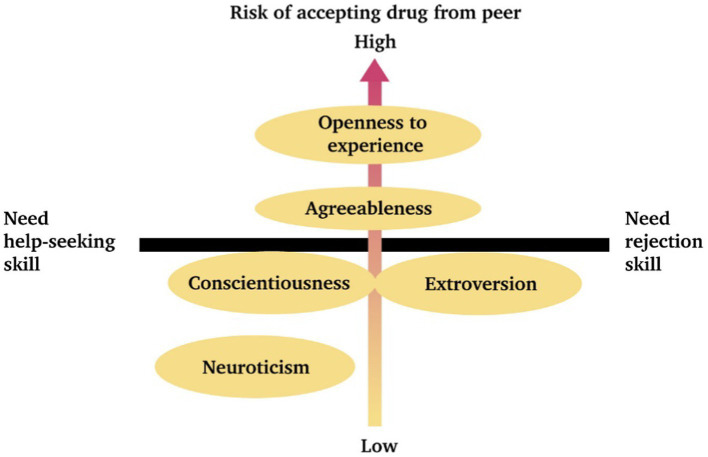
Adaptive anti-drug education framework.

To summarize, cognitive performance on the paper test was relatively low for respondents with neuroticism. In terms of drug refusal in tempting situations, people who were extroverted and open were more likely to accept drugs when pressured by their peers. Adaptivity corresponding to users’ characters is a crucial element in the modern system (Virvou et al., 2012; Zhu et al., 2016). Hence, these findings can provide reference points for designing **adaptive anti-drug education**. We also proposed a framework (Figure 11) for educators to “initially identify” the risk of accepting drug temptations and the direction of anti-drug strategies based on their personality traits.

In future research, the system could provide different supplementary materials to follow up with students’ system usage according to their personalities. For higher levels of automation, we could refer to the practice of these three papers about user modeling (Troussas et al., 2013, 2015; Guo et al., 2018) and employ algorithms for modeling social media user characteristics and to generate user clusters based on personal factors. In addition, teachers could harness different instructional strategies in accordance with the students’ personality traits. For instance, a teacher could separate the class into several homogeneous groups based on their personalities and then provide them with different learning tasks, such as more cognitive materials and explanation for neuroticism, materials for being extroverted, and openness to experiences, highlighting the cultivation of a refusal attitude and skills.

Two research limitations are found in the present study. The first one is that due to the complex experimental process and system deployment, this study cannot conduct experimental research on large-scale samples. Thus, there were inferencing limitations. The second research limitation is that in this study, the learners participated in the reading of the concept map rather than drawing. Whether such learning limitation affects the learning effect needs further research to explore. The students could draw their own in the future to indicate the effectiveness of their internalization.

## Data Availability Statement

The raw data supporting the conclusions of this article will be made available by the authors, without undue reservation.

## Ethics Statement

Ethical review and approval was not required for the study on human participants in accordance with the local legislation and institutional requirements. Written informed consent to participate in this study was provided by the participants’ legal guardian/next of kin.

## Author Contributions

T-CH initiated the study, conceived and planned the experiments with support from Y-JC, designed the serious game to collect the experimental data, and took the lead in writing the manuscript. Y-JC carried out the lag sequential analysis and interview, aided in interpreting the results, and worked on the manuscript. All authors provided critical feedback and helped to shape the research, analysis and manuscript. All authors contributed to the article and approved the submitted version.

## Funding

The study received funding from the Ministry of Science and Technology that is the government ministry of the Republic of China (Taiwan; MOST 109-2511-H-025-005-MY3 and MOST 108-2628-H-025-001-MY3).

## Conflict of Interest

The authors declare that the research was conducted in the absence of any commercial or financial relationships that could be construed as a potential conflict of interest.

## Publisher’s Note

All claims expressed in this article are solely those of the authors and do not necessarily represent those of their affiliated organizations, or those of the publisher, the editors and the reviewers. Any product that may be evaluated in this article, or claim that may be made by its manufacturer, is not guaranteed or endorsed by the publisher.
